# Supplementary material for: MetaQTL: a package of new computational methods for the meta-analysis of QTL mapping experiments
Source: BMC Bioinformatics. 2007 Feb 8;8:49. doi: 10.1186/1471-2105-8-49 (PMC1808479; doi:10.1186/1471-2105-8-49)
Supplement: Additional File 7 — MetaQTL Package : jar file and tutorial. This Zip archive contains both the MetaQTL JAR file and the files of the tutorial. [file 1471-2105-8-49-S7.zip › org.inra.metaqtl/doc/org/metaqtl/bio/entity/BioEntityContainer.html]

BioEntityContainer


|  |  |  |
| --- | --- | --- |
| **Overview** \| **Package** \| **Class** \| **Use** \| **Tree** \| **Deprecated** \| **Index** \| **Help** | |  |
| **PREV CLASS**   **NEXT CLASS** | **FRAMES**    **NO FRAMES**     **All Classes** |  |
| SUMMARY: NESTED \| FIELD \| CONSTR \| METHOD | DETAIL: FIELD \| CONSTR \| METHOD |  |


---


## org.metaqtl.bio.entity Class BioEntityContainer

```
java.lang.Object
  org.metaqtl.bio.entity.BioEntity
      org.metaqtl.bio.entity.BioEntityContainer
```

**All Implemented Interfaces:**: IBioAdaptable, IBioEntity

**Direct Known Subclasses:**: AlleleContainer, GroupContainer, Individual, IndividualContainer, LocusContainer

---

``` public abstract class BioEntityContainer extends BioEntity implements IBioEntity ```

Class Description Here

**Author:**
:   Jean-Baptiste Veyrieras

---

| **Field Summary** | |
| --- | --- |
| `protected  java.util.HashMap` | `entities` |

**Fields inherited from class org.metaqtl.bio.entity.BioEntity**

`name, parent, properties`


**Constructor Summary**

`BioEntityContainer()`

`BioEntityContainer(java.lang.String name,
IBioEntity parent)`


| **Method Summary** | |
| --- | --- |
| `protected  void` | `addEntity(IBioEntity entity)` |
| `protected  IBioEntity[]` | `entities()` |
| `protected  int` | `entityNumber()` |
| `protected  IBioEntity` | `getEntity(java.lang.String name)` |
| `protected  void` | `removeEntity(java.lang.String name)` |

**Methods inherited from class org.metaqtl.bio.entity.BioEntity**

`getName, getParent, getProperties, getType, newBioEntity, setName, setProperties`

**Methods inherited from class java.lang.Object**

`clone, equals, finalize, getClass, hashCode, notify, notifyAll, toString, wait, wait, wait`

**Methods inherited from interface org.metaqtl.bio.IBioEntity**

`getName, getParent, getProperties, getType, setName, setProperties`

**Methods inherited from interface org.metaqtl.bio.IBioAdaptable**

`getBioAdapter`

**Field Detail**

### entities

```
protected java.util.HashMap entities
```


**Constructor Detail**

### BioEntityContainer

```
public BioEntityContainer()
```

---


### BioEntityContainer

```
public BioEntityContainer(java.lang.String name,
                          IBioEntity parent)
```

**Parameters:**: `name` -: `parent` -


**Method Detail**

### addEntity

```
protected void addEntity(IBioEntity entity)
```

---


### getEntity

```
protected IBioEntity getEntity(java.lang.String name)
```

---


### entityNumber

```
protected int entityNumber()
```

---


### entities

```
protected IBioEntity[] entities()
```

---


### removeEntity

```
protected void removeEntity(java.lang.String name)
```


---


|  |  |  |
| --- | --- | --- |
| **Overview** \| **Package** \| **Class** \| **Use** \| **Tree** \| **Deprecated** \| **Index** \| **Help** | |  |
| **PREV CLASS**   **NEXT CLASS** | **FRAMES**    **NO FRAMES**     **All Classes** |  |
| SUMMARY: NESTED \| FIELD \| CONSTR \| METHOD | DETAIL: FIELD \| CONSTR \| METHOD |  |


---
